# Antibody seroprevalence against SARS-CoV-2 within the Canton of Sarajevo, Bosnia and Herzegovina—One year later

**DOI:** 10.1371/journal.pone.0265431

**Published:** 2022-03-31

**Authors:** Jasminka Prguda-Mujic, Osman Hasanic, Larisa Besic, Adna Asic, Sabina Halilovic, Aida Kulo Cesic, Neira Ljevakovic, Fildesa Muminovic, Sukrija Huseinovic, Daria Ler, Lana Salihefendic, Rijad Konjhodzic, Dragan Primorac, Damir Marjanovic

**Affiliations:** 1 Eurofarm Molecular Diagnostics Laboratory, Eurofarm Centre, Sarajevo, Bosnia and Herzegovina; 2 Department of Genetics and Bioengineering, International Burch University, Sarajevo, Bosnia and Herzegovina; 3 Department of Pharmacology, Clinical Pharmacology and Toxicology, Medical Faculty, University of Sarajevo, Bosnia and Herzegovina; 4 Alea Genetic Centre, Health Institute Alea Dr. Kandić, Sarajevo, Bosnia and Herzegovina; 5 St. Catherine Specialty Hospital, Zagreb/Zabok, Croatia; 6 Eberly College of Science, The Pennsylvania State University, University Park, State College, Pennsylvania, United States of America; 7 The Henry C. Lee College of Criminal Justice and Forensic Sciences, University of New Haven, West Haven, Connecticut, United States of America; 8 Medical School, University of Split, Split, Croatia; 9 School of Medicine, Faculty of Dental Medicine and Health, Josip Juraj Strossmayer University Osijek, Osijek, Croatia; 10 School of Medicine, Josip Juraj Strossmayer University Osijek, Osijek, Croatia; 11 Faculty of Medicine, University of Rijeka, Rijeka, Croatia; 12 Medical School REGIOMED, Coburg, Germany; 13 Institute for Anthropological Research, University of Zagreb, Zagreb, Croatia; Qatar University, QATAR

## Abstract

**Background:**

Serostudies are important resources when following pandemics and predicting their further spread, as well as determining the length of protection against reinfection and vaccine development. The aim of this study was to update data on the prevalence of seropositive individuals in Canton Sarajevo, Bosnia and Herzegovina (B&H) from September 2020 to May 2021.

**Methods:**

Anti-SARS-CoV-2 antibodies were quantified using an electrochemiluminescence immunoassay.

**Results:**

Compared to the period April–July 2020, when anti-SARS-CoV-2 antibodies were detected in 3.77% of samples, one year later (May 2021) the estimated percentage within the same population of the urban Canton Sarajevo was 29.9% (5,406/18,066). Of all anti-SARS-CoV-2 Ig-positive individuals, 53.27% were men, and 69.00% were of 50 years of age or younger. Also, the current update found the individuals 50 years of age or younger to be more frequently anti-SARS-CoV-2 Ig positive compared to older individuals. On the other hand, higher median anti-SARS-CoV-2 Ig levels were found in individuals > 50 years old than in younger individuals, as well as in men compared to women. Seropositivity gradually increased from September 2020 to May 2021, with the lowest frequency of positive cases (3.5%) observed in September 2020, and the highest frequency (77.7%) in January 2021.

**Conclusion:**

Our results provided important seroprevalence data that could help in planning restrictive local public health measures to protect the population of Sarajevo Canton, especially considering that at the time of the study the vaccines were virtually inaccessible to the general population not belonging to any of the high-priority groups for vaccination.

## Introduction

The coronavirus disease 2019 (COVID-19) has resulted in more than 192 million cases of confirmed infection, and more than 4.2 million deaths worldwide as of week 27 update July 15, 2021 [1]. The actual prevalence number of infections is believed to be significantly higher in comparison with Real-time-PCR (qRT-PCR)-confirmed cases. In Bosnia and Herzegovina (B&H), 134 thousand qRT-PCR-confirmed cases and 5.5 thousand deaths have been documented, whilst in the Canton Sarajevo, 46,571 qRT-PCR-confirmed cases have been acknowledged [2].

Serological testing for COVID-19 is becoming increasingly important within the research community and immunity surveillance efforts. Likewise, seroprevalence studies are crucial for estimating the infection, prevalence, and death rate, as well the spread of the virus in the population-based on disease recovery and vaccination. Henceforward, seroprevalence will become a parameter that will aid in planning and monitoring the impact of implementation and relaxation of epidemic restrictions.

Serological testing is performed on whole blood, serum, or plasma samples via a relatively simple and rapid procedure, requiring less expertise and simpler laboratory settings compared to molecular methods. Tests are designed to detect either total immunoglobulins (Ig) or to differentiate between immunoglobulins M (IgM) and immunoglobulins G (IgG) fractions [3, 4]. Previous studies have reported 77.3% sensitivity and 100% specificity for IgM, and 83.3% sensitivity, and 95% specificity for IgG [5]. Serological assays directed towards SARS-CoV-2 are based on the detection of antibodies against a 2-subunit viral spike (S) protein with S1 being responsible for receptor binding and S2 for fusion [3, 6].

In the second year of the COVID-19 pandemic seroprevalence studies are gaining considerable attention. Detection of antibodies is an important resource in controlling the epidemiological situation, determining the duration of protection against reinfection, developing vaccines and therapeutic monoclonal antibodies, as well as for forecasting the future of the pandemic. In addition, it was shown that the number of individuals with antibodies against SARS-CoV-2 may be 10-fold higher than the number of reported cases [7]. On the other hand, serosurveys are criticized for two main limitations, namely design bias and inadequate testing methods, both of which are likely to lead to overestimating the number of seropositive individuals [8].

Several early seroprevalence studies were already conducted within different countries, including B&H [9–19].

The aim of the present study was to update data on the prevalence of seropositive individuals in Canton Sarajevo, B&H, from September 2020 to May 2021, to determine the time frame for the peak frequency of positive results and to compare our results with those published by the official authorities and governmental bodies.

## Materials and methods

### Participants

The research was conducted at Eurofarm Central Laboratory, from September 2020 to May 2021. All participants signed written informed consent (prepared in accordance with the principles of the Declaration of Helsinki), reviewed and approved by the local ethics committee [20].

### Materials

Peripheral blood samples and nasopharyngeal swabs were collected. Blood samples were collected by venipuncture in Vacusera vacutainers with CAT serum (BD Vacutainer^®^, Germany). Samples were transferred for centrifuge at 500xg for 10 minutes and analyzed the same day.

### Methods

#### ECLIA

The serum samples were analyzed using CE.IVD Roche CobasElecsys^®^ Anti-SARS-CoV-2, an electrochemiluminescence immunoassay (ECLIA), for the qualitative detection of total pan-Ig, IgG, IgM, and IgA (Roche Diagnostics, Rotkreuz, Switzerland). The test was performed according to manufacturer instructions (Roche Diagnostics, Rotkreuz, Switzerland). The test applies the recombinant nucleocapsid (N) protein of SARS-CoV-2 as an antigen and the method is based on the”Double-antigen sandwich”format between the donor’s plasma, biotinylated SARS-CoV-2 specific N antigen, and SARS-CoV-2-specific recombinant N antigen labeled with a ruthenium complex. Streptavidin-coated microparticles are added after concomitant incubation of reagents. The immune-complex is bound to the solid phase via biotin-streptavidin interaction and extracted for cell measuring through a Roche Cobas E411 Analyzer, magnetically capturing microparticles on the surface of the electrode. Materials that remain unbound are washed away, and the emission levels of induced chemiluminescence by a specific electrical current on an electrode are measured with a photomultiplier.

Results are generated with an interpolating ECLIA signal with a threshold produced during calibration. The result is given either as reactive or non-reactive in the form of a cutoff index (COI; signal sample/cutoff). If the COI is < 1.0 then the result is considered non-reactive (negative for anti-SARS-CoV-2 antibodies); if COI is ≥ 1.0 then the result is considered reactive (positive for anti-SARS-CoV-2 antibodies). Elecsys^®^Anti-SARS-CoV-2 exhibits a high overall clinical specificity of 99.8%, and sensitivity from 60.2–99.5% (Roche Diagnostics, Rotkreuz, Switzerland) with a lack of cross-reactivity to the common cold coronaviruses according to the manufacturer’s package insert.

#### ELISA

The ELISA method was used for testing the control group (n = 60) samples, selected randomly from the tested cohort. ELISA was performed using a commercial ELISA kit based on recombinant spike glycoprotein (S) and nucleocapsid protein (N) antigens of SARS-CoV-2 (ELISA COVID-19 IgG; Vircell Microbiologists, Granada, Spain). Results were expressed through the antibody index; AI = (sample OD/cut off serum mean OD) × 10, and interpreted as follows: IgG < 4, negative; 4–6, borderline; > 6, positive.

#### qRT-PCR

Real-time PCR was applied for the analysis of nasopharyngeal swab samples of the negative control group (n = 20). RNA was isolated using Bosphore^®^ EX-Tract Dry Swab RNA Solution (Anatolia Geneworks, Istanbul, Turkey), and the following kit was implemented for the detection of SarS-CoV-2 [Bosphore^®^ Novel Coronavirus (2019-nCoV) detection kit V2]. The SARS-CoV-2 kit detects two regions of the virus in a single reaction: the E gene is used for screening purposes whereby the orf1ab target region is used to discriminate SARS-CoV-2 specifically. Applied Biosystems QuantStudio 5 Real-Time PCR System [Thermo Fisher Scientific Inc. (NYSE:TMO), Waltham, Massachusetts, USA] was applied for detection.

### Statistical analysis

The results were expressed in the form of COI. The chi-square test was deployed when testing the association between the proportion of SARS-CoV-2 positive cases and gender, and between individuals of 50 years of age or younger and > 50 years old at the time of testing. The Mann-Whitney U test was used to compare the full distribution of ranks of anti-SARS-CoV-2 Igs levels between men and women who tested positive, and between individuals of 50 years of age or younger and > 50 years old at the time of testing, all of whom tested positive. The two-sample t-test was employed to compare mean ages between men and women who tested positive. Binary logistic regression analysis was performed to assess independent predictors of SARS-CoV-2 positive cases. The variables tested as covariates were age and gender. Data analysis was performed in MS Excel, as described by McDonald in 2014 [21], and IBM SPSS Statistics for Windows v27 (IBM, Armonk, NY).

## Results

A total of n = 18,066 individuals were tested in the period from September 2020 to May 2021. Prior to testing and analyzing, 60 control samples, selected randomly from the tested cohort, have been used for verification and correlation between ECLIA and ELISA methods, 30.4% were positive during ECLIA tests, whilst ELISA tests gave 27.2% positive results. The fractions of positive results did not differ significantly between the two methods (p = 0.41). The nasopharyngeal samples (n = 20) that tested negative on qRT-PCR disclosed negative results on both ECLIA and ELISA.

### Demographic data

Mean ± standard deviation of age at testing was 42.25 ± 15.27 years, ranging from minimum 0 to maximum 93 years, with 70.7% of the participants being 50 years of age or younger, and the remaining 29.3% were > 50 years old. A total of 54.2% of the study participants were men, while the remaining 45.8% were women.

### SARS-CoV-2 positive cases

An individual was considered to be positive for anti-SARS-CoV-2 Ig if COI was ≥ 1.0. Based on this cutoff level, a total of 5,406 individuals tested positive, which accounts for 29.92%. The mean age among individuals who tested positive was 43.21 ± 14.91 years, and the median anti-SARS-CoV-2 Ig level was 18.9; interquartile range (IQR) 43.9 (Table 1).

**Table 1:** The proportion of positive cases and the effect of age and gender.

		Variables		P value		
	Positive cases, n (%)	Age (years)	Anti-SARS-CoV-2 Ig level	Two sample t-test, age	Mann-Whitney U test, anti-SARS-CoV-2 Ig	Chi-square test, anti-SARS-CoV-2 Ig
**Total cases n = 18,066**	5,406 (29.92)	43.21 ± 14.91	18.9 (43.9)			
**Male**	2,880 (53.27)	43.51 ± 14.99	20.4 (49.73)	0.112	**p < 0.001**	0.090
**Female**	2,526 (46.72)	42.86 ± 14.82	17.3 (38.70)			

Data are presented by frequencies (%), by mean ± standard deviation or by median (IQR). P values that were lower than the 0.05 critical level shown in bold.

### Seroprevalence according to age

**Fig 1:**
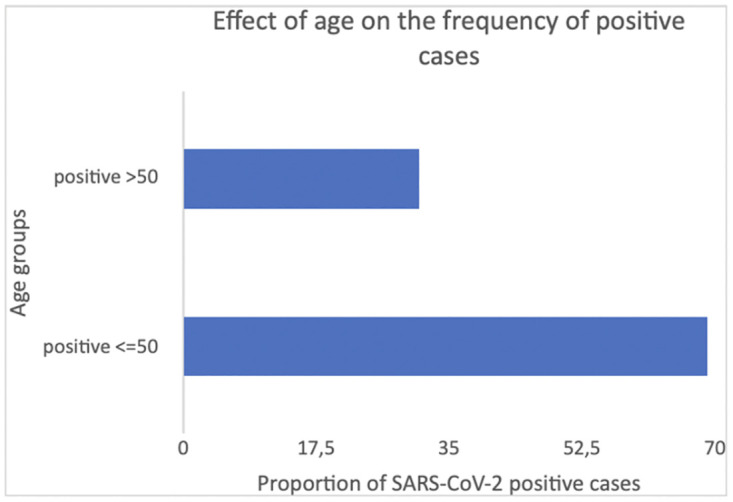
Effects of age on the frequency of positive cases. A total of 69.02% of the individuals who tested positive were of 50 years of age or younger, while 30.98% were > 50 years old, and the chi-square test revealed this association between the proportion of positive cases and two age groups to be significant (*X*^*2*^ = 10.274; df = 1; p = 0.001).

A total of 3731 (69.00%) of positive individuals were of 50 years of age or younger, while 1675 (31.00%) were > 50 years old. The chi-square test revealed this association between the proportion of positive cases and two age groups to be significant (*X*^*2*^ = 10.274; df = 1; p = 0.001; Fig 1).

**Fig 2:**
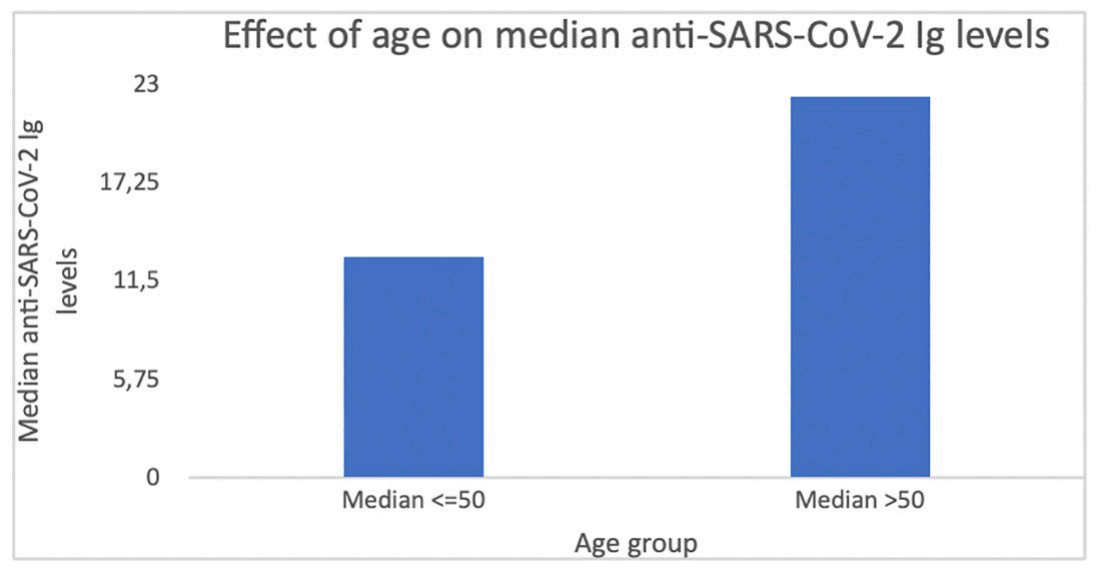
Effect of age on median anti-SARS-CoV-2 Ig levels. Significantly higher median anti-SARS-CoV-2 Ig level was found in the individuals > 50 years old when compared to the younger group (25.9; IQR 59.00 vs 16.00, IQR 36.00; U = 2535473.00; p < 0.001).

Significantly higher median anti-SARS-CoV-2 Ig level was found in individuals >50 years of age when compared to the younger group (25.9; IQR 59.00 vs 16.00, IQR 36.00; U = 2535473.00; p < 0.001; Fig 2).

### Seroprevalence according to gender

**Fig 3:**
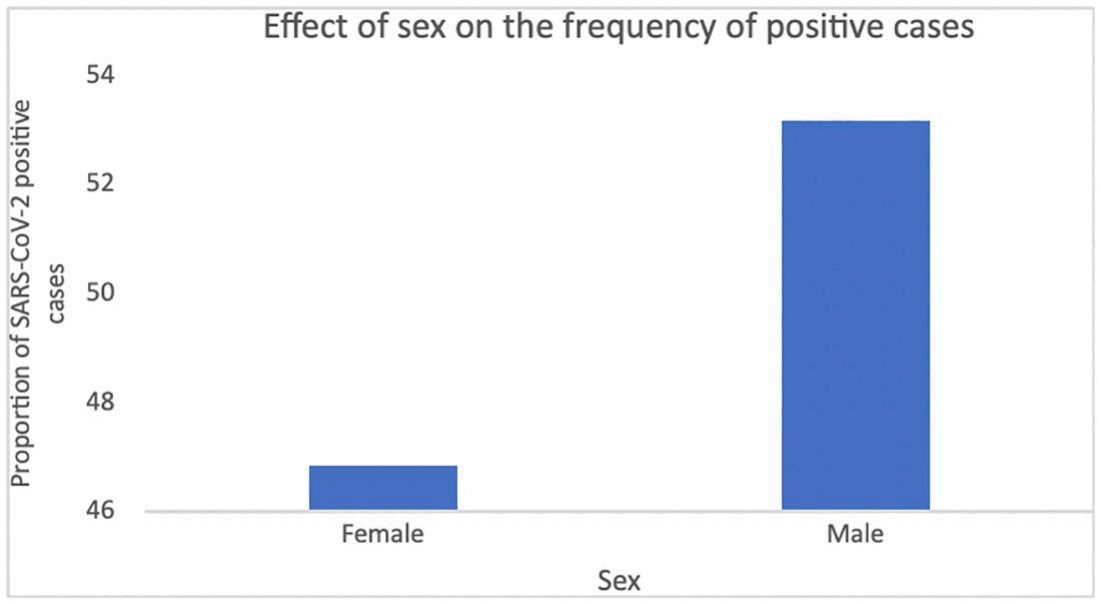
Effects of sex on the frequency of positive cases. A total of 2,880 (53.27%) anti-SARS-CoV-2 Ig-positive individuals were men, while 2,526 (46.72%) were women, the chi-square test revealed no significant association between the proportion of positive cases and gender (*X*^*2*^ = 2.890; df = 1; p = 0.090).

A total of 2,880 (53.27%) anti-SARS-CoV-2 Ig-positive individuals were men, while 2,526 (46.72%) were women, and the chi-square test revealed no significant association between the proportion of positive cases and gender (*X*^*2*^ = 2.890; df = 1; p = 0.090; Table 1; Fig 3).

**Fig 4:**
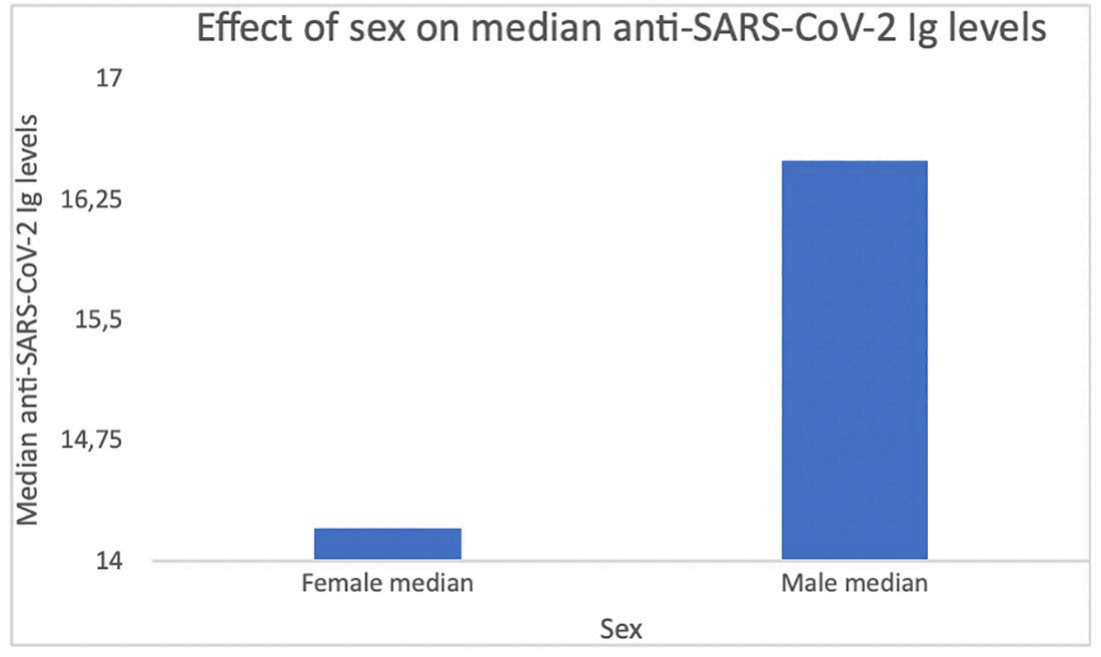
Effects of sex on median anti-SARS-CoV-2 Ig levels. The median anti-SARS-CoV-2 Ig level among individuals who tested positive was 18.9, interquartile range 43.9. Significantly higher median anti-SARS-CoV-2 Ig level was found in men when compared to women (20.40, IQR 49.72 vs 17.3, IQR 38.7; U = 3365817.50; p < 0.001).

Significantly higher median anti-SARS-CoV-2 Ig level was found in men when compared to women (20.40, IQR 49.72 vs 17.3, IQR 38.7; U = 3365817.50; p < 0.001; Table 1; Fig 4).

**Fig 5:**
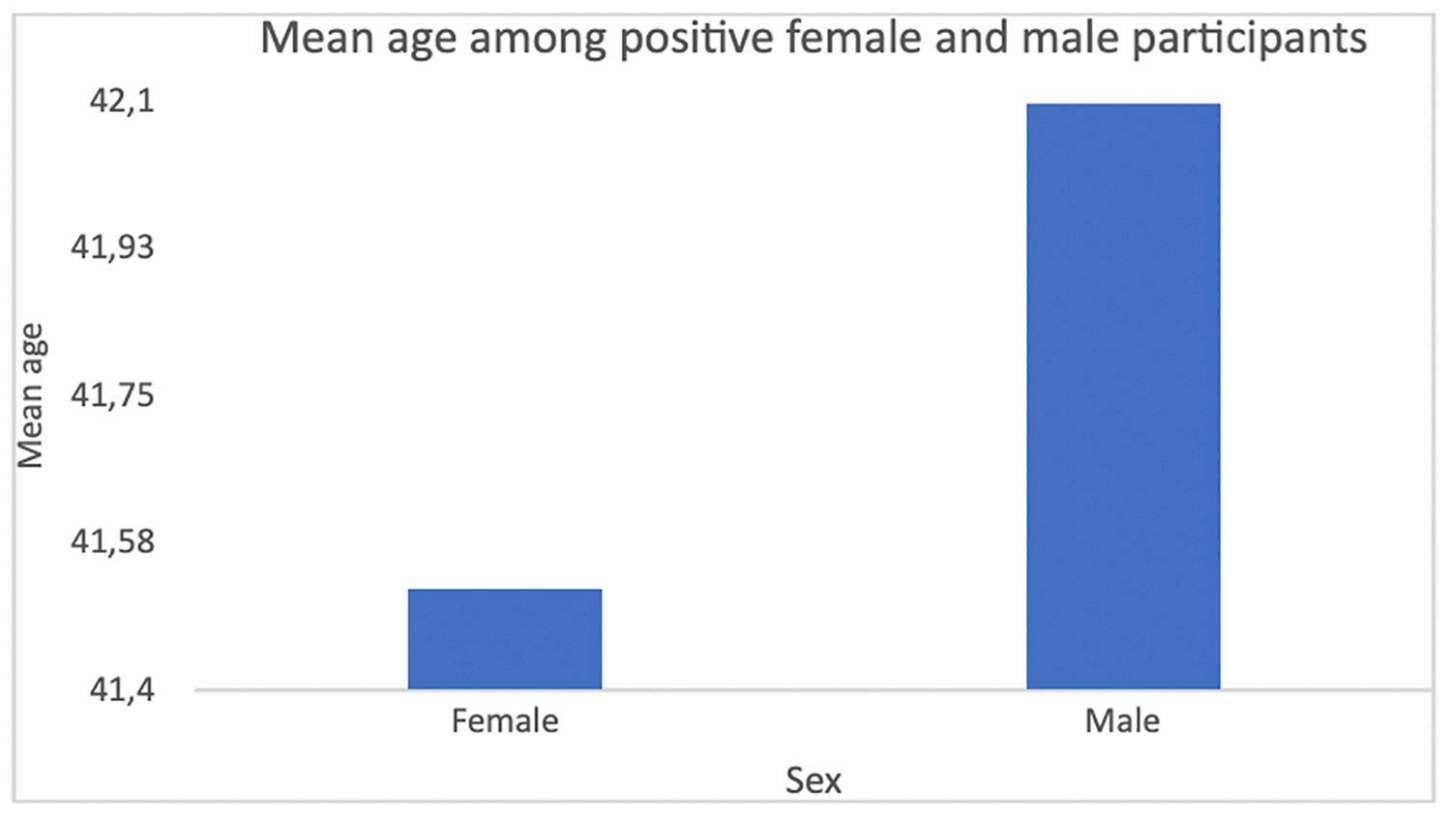
Mean age among positive female and male participants. The mean age of all individuals who tested positive was 43.21 ± 14.91. Mean age did not differ significantly between men (43.51 ± 14.99) and women (42.86 ± 14.82) who tested positive (t test = 1.589; p = 0.112).

The mean age did not differ significantly between men (43.51 ± 14.99) and women (42.86 ± 14.82) who tested positive (Two-sample t-test = 1.589; p = 0.112; Table 1; Fig 5).

In binary logistic regression analysis, the odds ratios for age categorised into 10-year age bands were insignificant, while individuals of 50 years of age or younger were, independently of gender, more likely to have positive anti-SARS-CoV-2 Ig levels compared with individuals age > 50 years. The unadjusted and adjusted odds ratio for two age groups and gender are shown in Table 2.

**Table 2:** Binary logistic regression analysis of independent predictors of positive anti-SARS CoV-2 Ig levels.

Covariates	Odds ratio	95% CI	p-value
**Age** *	0.893	0.833–0.957	**0.001**
*The model was not statistically significant X*^*2*^ *= 10*.*183*, *p = 0*.*001; it explained 0*.*1% (Nagelkerke R*^*2*^*) of the variance and correctly classified 70*.*1% of cases*.			
**Gender**	0.946	0.888–1.009	0.089
*The model was not statistically significant X*^*2*^ *= 2*.*886*, *p = 0*.*089; it explained 0*.*0% (Nagelkerke R*^*2*^*) of the variance and correctly classified 70*.*1% of cases*.			
**Age** *	0.891	0.832–0.955	**0.001**
**Gender**	0.943	0.885–1.005	0.073
*The model was not statistically significant X*^*2*^ *= 13*.*398*, *p = < 0*.*001; it explained 0*.*1% (Nagelkerke R*^*2*^*) of the variance and correctly classified 70*.*1% of cases*.			

*two age categories: 50 years of age or younger and > 50 years of age.

### The frequency of positive cases throughout a nine-month time frame

**Fig 6:**
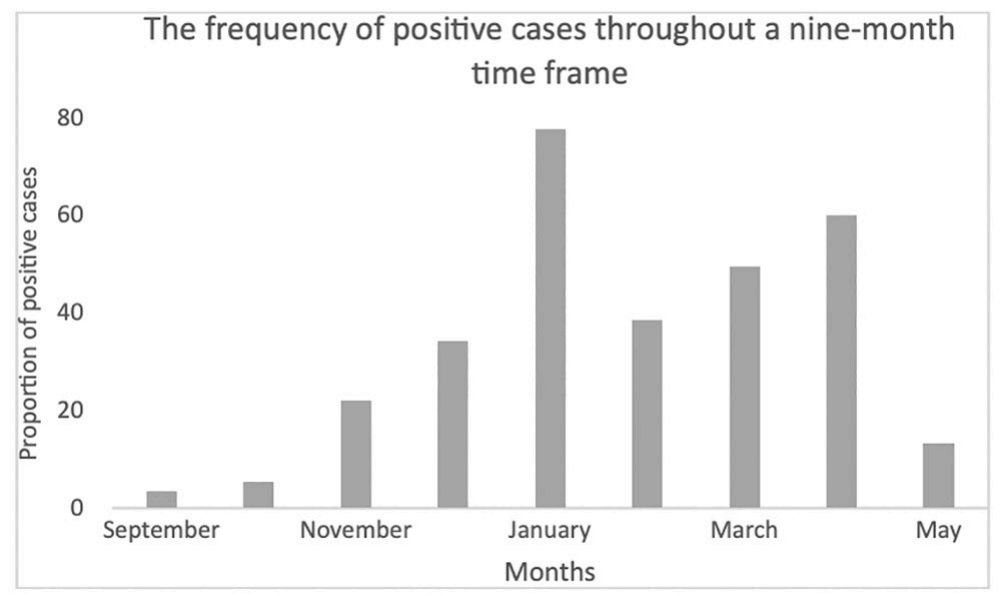
The frequency of positive cases throughout a nine-month time frame. The lowest frequencies of positive cases were observed in September 2020 (3.5%, 84/2,376) and October (5,4%, 82/1,509) 2020 while the highest frequencies were observed in January 2021 (77.7%, 483/622), and in April 2021 (60.1%, 1,042/1,734).

Fig 6 shows the frequency of seropositive cases detected for each month separately for the same nine-month period covered in this study.

The lowest frequencies of positive cases were observed in September 2020 (3.5%, 84/2,376) and October (5,4%, 82/1,509) 2020 while the highest frequencies were observed in January 2021 (77.7%, 483/622), and in April 2021 (60.1%, 1,042/1,734) (Fig 6).

## Discussion

The present study provides a year-later update on the first population-wide serosurvey in B&H, i.e. an update on the anti-SARS-CoV-2 antibody prevalence in Canton Sarajevo. A significantly increased number of seropositive individuals during the one-year period of COVID-19 pandemics was reported. In a previously published seroprevalence study for the urban Canton Sarajevo region, 2,841 samples were collected between April and July 2020, and anti-SARS-CoV-2 antibodies were detected in 3.77% of samples [12]. Our latest dana revealed that one year later (May 2021), the estimated percentage within the same population was actually 29.9%. Also, the current update found the individuals 50 years of age or younger to be more frequently SARS-CoV-2 positive compared to older individuals. On the other hand, higher median anti-SARS-CoV-2 Ig levels were found in individuals > 50 years old than in younger individuals, as well as in men compared to women.

In terms of the incidence of infection cases, lower figures were recorded in B&H at the beginning of the outbreak when compared to the rest of Europe [22], most probably due to the country, and Sarajevo Canton especially, being under a strict lockdown from mid-March until mid-May 2020. A subsequent visible increase in the number of seropositive individuals may be due to non-selective and non-systematic lifting of a whole set of epidemiological measures, such as relaxing of obligatory physical distancing practices, allowing mass gatherings, liberal rules for border crossing, as well as confirmation of new viral genetic variants (e.g., B1.1.7), increased testing capacity, and local transmission of the virus [13, 22]. It is also important to emphasize that the vaccination campaign was almost nonexistent in B&H during that time of this study, that is, until May 2021.

Previously reported general population seroprevalence of anti-SARS-CoV-2 antibodies varies across different regions and countries [9]. Early seroprevalence studies were conducted as early as April 2020. Regarding studies for populations neighboring B&H, a study was conducted on a sample of 1,494 factory workers in the counties of Split-Dalmatia and Šibenik-Knin in Croatia in April 2020. Antibodies were detected in 1.27% of the total number of study participants, thus further exemplifying the trend of low seropositivity in the first phase of the pandemics [19]. In general, the frequency of seropositive individuals varies from < 0.1% to more than 20% in different studies depending on the country and implemented measures, sampling, and testing method, and phase of pandemics in which studies were executed [26, 27]. On the other hand, the latest regional dana for the Western Balkans report a seropositivity of around 17% for Northern Serbia (Vojvodina region) in September 2020 [13].

Although seropositive individuals were detected in all age groups, a significant difference in seropositivity among different age groups was noted in the present study. Similar to the Northern Serbian population [13], it seems that the younger population group exhibited a higher frequency of seropositive cases due to their behavioral habits, including the lack of physical distancing, traveling, and not following the proposed epidemiological measures. While the results from the Northern Serbian population suggested women to be more exposed compared to men [13], we were only able to estimate no difference in proportion of infected men and women.

The current official Sarajevo Canton data is reporting 52,752 qRT-PCR-positive cases [2], which corresponds to approximately 12% of the current population, according to the 2013 Census [23]. On the country level, there are 205,000 qRT-PCR-confirmed cases in B&H [24], which represents around 6% of the total population according to the same Census [23]. When comparing the peaks in the number of detected cases, the first peak reported from molecular data in the second phase of pandemics was in mid-November 2020, while the first peak based on antibody testing was recorded in January 2021. Such findings are generally expected considering the time gap between the active infection and seroconversion. The second peak for both molecular and serological testing was reported in April 2021 (Fig 6), which points out the fact that the citizens of the Sarajevo Canton relied more on serological testing. Likewise, another regional study aiming to assess the prevalence and dynamics of anti-SARS-CoV-2 IgA and IgG antibodies in the cohort of asymptomatic individuals estimated the frequency of qRT-PCR negative individuals to approximately 5.9–10.5%. Despite this, their exposure to SARS-CoV-2 during the pandemic could not be excluded [25].

These results can help in estimating the risk rates for virus spread, virus infection, the likelihood of reaching herd immunity, and prioritizing vaccine recipient groups. The threshold level of seropositive individuals for reaching herd immunity for COVID-19 is estimated to be approximately 70% [26], and maybe even higher due to variant B.1.617.2, classified as a variant of concern by the Center for Disease Control and Prevention [27].

Although this study represents the year-later update on the first population-wide serosurvey in B&H, it has several limitations. Firstly, we collected the blood samples from all the patients who came for serological testing, without collecting data regarding the reasons for testing, Covid-19 history or severity of their illness, comorbidities. Thus, we were not able to analyse if in subjects with the previous infection with COVID-19, e.g. more than 6 months previously, serum antibody levels may have decreased to low levels. Also, we could not analyse if vaccination affected the antibody titers as the massive vaccination was not available at the time of our study. Secondly, we provided 60 control samples, selected randomly from the tested cohort, while the inclusion of serum samples from at least some of the individuals before the emergence of SARS-CoV-2 would be a better option. Thirdly, the updated data from other countries, except their specific subpopulation, i.e. children, pregnant women, healthcare workers, specific groups of patients such as patients on haemodialysis, patients with autoimmune diseases, etc., lack, being the major limitation of our discussion section. Finally, interpretation of serologic data may be challenging. i.e. the ideal scenario would include the collection of paired sera that would give a better estimate of the rate of newly acquired infection in the area over time.

Our results provided important seroprevalence data that could help in planning restrictive local public health measures to protect the population of Sarajevo Canton, especially considering that at the time of the study the vaccines were virtually inaccessible to the general population not belonging to any of the high-priority groups for vaccination. Thus, while expecting the next COVID-19 waves of pandemics to occur in the near future within our population, new prospective seroprevalence studies should include measurements of other types of SARS-CoV-2 antibodies such as neutralising and vaccine-induced ones.

## Supporting information

S1 Data (XLSX)

## References

[1] Covid-19 situation update worldwide. Center for Disease Control and Prevention. https://www.ecdc.europa.eu/en/geographical-distribution-2019-ncov-cases. [accessed July 20, 2021].

[2] Public Health Department of the Sarajevo Canton. 2021. https://zzjzks.ba/ [accessed July 21, 2021].

[3] Krammer F, Simon V. Serology assays to manage COVID-19. Science. 2020;368(6495):1060–1061. doi: 10.1126/science.abc1227 . 32414781

[4] Loeffelholz MJ, Tang YW. Laboratory diagnosis of emerging human coronavirus infections—the state of the art. Emerg Microbes Infect. 2020;9(1):747–756. doi: 10.1080/22221751.2020.1745095 . 32196430 PMC7172701

[5] Xiang F, Wang X, He X, Peng Z, Yang B, Zhang J, et al. Antibody Detection and Dynamic Characteristics in Patients With Coronavirus Disease 2019. Clin Infect Dis. 2020;71(8):1930–1934. doi: 10.1093/cid/ciaa461 . 32306047 PMC7188146

[6] Tang YW, Schmitz JE, Persing DH, Stratton CW. Laboratory Diagnosis of COVID-19: Current Issues and Challenges. J Clin Microbiol. 2020;58(6):e00512–20. doi: 10.1128/JCM.00512-20 . 32245835 PMC7269383

[7] Stephens DS, McElrath MJ. COVID-19 and the Path to Immunity. JAMA. 2020;324(13):1279–1281. doi: 10.1001/jama.2020.16656 . 32915201 PMC12177933

[8] Kadkhoda K. Letter to the editor: COVID-19: how accurate are seroprevalence studies? Euro Surveill. 2020;25(30):2001374. . 32734860 10.2807/1560-7917.ES.2020.25.30.2001374 PMC7393853

[9] Eckerle I, Meyer B. SARS-CoV-2 seroprevalence in COVID-19 hotspots. Lancet. 2020;396(10250):514–515. doi: 10.1016/S0140-6736(20)31482-3 . 32645348 PMC7336129

[10] Tsitsilonis OE, Paraskevis D, Lianidou E, Pierros V, Akalestos A, Kastritis E, et al. Seroprevalence of Antibodies against SARS-CoV-2 among the Personnel and Students of the National and Kapodistrian University of Athens, Greece: A Preliminary Report. Life (Basel). 2020;10(9):214. doi: 10.3390/life10090214 . 32967110 PMC7555935

[11] Venugopal U, Jilani N, Rabah S, Shariff MA, Jawed M, Mendez Batres A, et al. SARS-CoV-2 seroprevalence among health care workers in a New York City hospital: A cross-sectional analysis during the COVID-19 pandemic. Int J Infect Dis. 2021;102:63–69. doi: 10.1016/j.ijid.2020.10.036 . 33075539 PMC7566823

[12] Ašić A, Prguda-Mujić J, Salihefendić L, Bešić L, Ler D, Čeko I, et al. Serological testing for SARS-CoV-2 in Bosnia and Herzegovina: first report. Arch Med Sci. 2021;17(3):823–826. doi: 10.5114/aoms/134143 . 34025854 PMC8130482

[13] Ristić M, Milosavljević B, Vapa S, Marković M, Petrović V. Seroprevalence of antibodies against SARS-CoV-2 virus in Northern Serbia (Vojvodina): A four consecutive sentinel population-based survey study. PLoS One. 2021;16(7):e0254516. doi: 10.1371/journal.pone.0254516 . 34242377 PMC8270141

[14] Pollán M, Pérez-Gómez B, Pastor-Barriuso R, et al. Prevalence of SARS-CoV-2 in Spain (ENE-COVID): a nationwide, population-based seroepidemiological study. Lancet. 2020;396(10250):535–544. doi: 10.1016/S0140-6736(20)31483-5 32645347 PMC7336131

[15] Public Health Agency Sweden. Första resultaten från pågående undersökning av antikroppar för covid-19-virus. May 20, 2020. https://www.folkhalsomyndigheten.se/nyheter-och-press/nyhetsarkiv/2020/maj/forsta-resultaten-fran-pagaende-undersokning-avantikroppar-for-covid-19-virus [accessed July 23, 2021].

[16] Stringhini S, Wisniak A, Piumatti G, et al. Seroprevalence of anti-SARS-CoV-2 IgG antibodies in Geneva, Switzerland (SEROCoV-POP): a population-based study. Lancet. 2020;396(10247):313–319. doi: 10.1016/S0140-6736(20)31304-0 32534626 PMC7289564

[17] Xu X, Sun J, Nie S, et al. Seroprevalence of immunoglobulin M and G antibodies against SARS-CoV-2 in China. Nat Med. 2020;26;1193–1195. doi: 10.1038/s41591-020-0949-6 32504052

[18] Song SK, Lee DH, Nam JH, Kim KT, Do JS, Kang DW, et al. IgG Seroprevalence of COVID-19 among Individuals without a History of the Coronavirus Disease Infection in Daegu, Korea. J Korean Med Sci. 2020;35(29):e269. doi: 10.3346/jkms.2020.35.e269 . 32715672 PMC7384903

[19] Jerković I, Ljubić T, Bašić Ž, Kružić I, Kunac N, Bezić J, et al. SARS-CoV-2 Antibody Seroprevalence in Industry Workers in Split-Dalmatia and Šibenik-Knin County, Croatia. J Occup Environ Med. 2021;63(1):32–37. doi: 10.1097/JOM.0000000000002020 . 32925526 PMC7773159

[20] World Medical Association. World Medical Association Declaration of Helsinki: ethical principles for medical research involving human subjects. JAMA. 2013; 310(20):2191–4. doi: 10.1001/jama.2013.281053 . 24141714

[21] McDonald JH. Handbook of Biological Statistics. 3rd ed. Sparky House Publishing, Baltimore, Maryland; 2014.

[22] Hukic M, Ponjavic M, Tahirovic E, Karabegovic A, Ferhatbegovic E, Travar M, et al. SARS-CoV-2 virus outbreak and the emergency public health measures in Bosnia and Herzegovina: January—July, 2020. Bosn J Basic Med Sci. 2021;21(1):111–116. doi: 10.17305/bjbms.2020.5081 . 33091331 PMC7861623

[23] The Census of the Population of Bosnia and Herzegovina. 2013. http://www.statistika.ba/ [accessed July 21, 2021].

[24] The Ministry of Civil Affairs of Bosnia and Herzegovina. 2021. http://mcp.gov.ba/Publication/Read/sluzbene-informacije-o-koronavirusu-covid-19?pageId=97 [accessed July 21, 2021].

[25] Hassanmirzaei B, Haratian Z, Ahmadzadeh Amiri A, Ahmadzadeh Amiri A, Moghadam N. SARS-CoV-2 serological assay and viral testing: a report of professional football setting. Postgrad Med J. 2021;postgradmedj-2021-140176. doi: 10.1136/postgradmedj-2021-140176 . 37066496

[26] Kontou PI, Braliou GG, Dimou NL, Nikolopoulos G, Bagos PG. Antibody Tests in Detecting SARS-CoV-2 Infection: A Meta-Analysis. Diagnostics (Basel). 2020;10(5):319. doi: 10.3390/diagnostics10050319 . 32438677 PMC7278002

[27] SARS-CoV-2 Variant Classifications and Definitions. Center for Disease Control and Prevention. 2021. https://www.cdc.gov/coronavirus/2019-ncov/variants/variant-info.html [accessed July 22, 2021].

